# Genetic manipulation of the mammary gland and potential applications

**DOI:** 10.18632/oncotarget.27044

**Published:** 2019-07-02

**Authors:** Hiroaki Tagaya, Kentaro Semba, Kosuke Ishikawa

**Affiliations:** ^1^ Japan Biological Informatics Consortium, Tokyo, Japan

**Keywords:** piggyBac, transposon, MaSC, transgenesis, electroporation

The development of genetically engineered mouse models such as transgenic or knock out mice has contributed to our deep understanding of the *in vivo* mechanisms of tumorigenesis and metastasis. However, this engineering approach is not practical for high-throughput and larger-scale gene evaluation. Recently, we reported a transposon-based genetic manipulation system in the mammary gland that can be applied to oncogene screening, lineage tracing, and marker gene screening [1].

The mammary gland, which is mainly composed of a bilayered ductal structure consisting of outer basal cells and inner luminal cells, is the characteristic organ in which most differentiation and development events occur after puberty. When any portion from the mammary epithelial tree is transplanted into epithelium-free mammary fat pads, the segment can regenerate the entire ductal mammary gland with milk-producing function. This indicated that repopulating cells are distributed throughout the mammary gland. Genetic manipulation by viral vectors and purification technologies with cell surface markers demonstrated that this repopulation activity is exerted by a single cell, which is inferred to be a mammary stem cell (MaSC) [2–4]. Subsequently, lenti- or retro-viral-based gene transfer into the MaSC-enriched fraction successfully generated genetic engineered mammary gland [5, 6]. However, there are intrinsic limitations in introducing long or complex sequences that include multiple promoters or transcription termination sequences owing to their viral packaging mechanism. To address this problem, we recently established another method using the *piggyBac* transposon system combined with electroporation [1]. The transposon system has almost no restrictions on sequence type or cargo length (> 200 kb BAC) [7], so that the design of complex and long DNA elements, including those for overexpression, knockout, or knockdown experiments, are almost limitless. In a study by Tagaya *et al*, two marker expression units (mCherry and luciferase) were introduced to monitor the remodeling and tumor progression of genetically modified mammary glands, along with a Tet-on system unit to allow inducible gene expression. There was no problem in the introduction of a transposon vector into MaSC-enriched fractions even if those DNA units were loaded on one vector, and inducible expression of polyoma middle T antigen (PyMT) with this system phenocopied the PyMT transgenic mouse [1]. This vector adopts the Gateway® system, which facilitates the preparation of a DNA library and oncogene screening. It would also be possible to establish a so-called gene trap system; this system could induce both gain-of-function and loss-of-function mutations by random integration of DNA elements with the combination of a promoter, a splicing acceptor/donor, and/or transcription termination sequences to screen oncogene and tumor suppressor genes simply by making some changes to the transposon vector.

Cell type-specific expression of a gene in target cells is one of the most useful and promising applications of our system. Breast cancer is classified into at least six distinct subtypes on the basis of gene expression profiling: claudin-low, basal-like, HER2^+^, luminal A, luminal B, and normal-like. It is believed, although this is still controversial, that distinct cells of origin may cause each subtype [8]. Lineage tracing technology is essential to track the progenies from the predicted cells of origin and to investigate their behavior and contribution to normal tissue and cancer development. For this purpose, well-characterized cell type-specific promoters have often been used to mark the cell types of interest. However, a marker for basal progenitors, an uncharacterized but a predicted critical cell for breast cancer development and malignancy, has not been reported thus far. The recently reported highly sensitive promoter trap technology [9] coupled with this genetic manipulation of the mammary gland may enable us to identify novel cell type-specific promoters.
